# Prevalence of chronic endometritis in infertile women undergoing hysteroscopy and its association with intrauterine abnormalities: A Cross-Sectional study

**DOI:** 10.5935/1518-0557.20240011

**Published:** 2024

**Authors:** Sedigheh Hosseini, Hajar Abbasi, Saghar Salehpour, Nasrin Saharkhiz, Mitra Nemati

**Affiliations:** 1 Preventative Gynecology Research Center, Shahid Beheshti University of Medical Sciences, Tehran, Iran

**Keywords:** chronic endometritis, *in-vitro* fertilization, hysteroscopy, intrauterine abnormalities

## Abstract

**Objective:**

Chronic endometritis (CE) is an inflammatory condition with several different risk factors. We aimed to examine whether intrauterine abnormalities, such as endometrial polyps, submucosal myomas, intrauterine adhesions, or a septate uterus, were associated with an increased likelihood of developing chronic endometritis.

**Methods:**

A cross-sectional study was conducted on 335 infertile women who underwent hysteroscopy surgery at the Ayatollah Taleghani Hospital Infertility Center, affiliated by Shahid Beheshti University of Medical Sciences, in 2022. All participants in the study underwent hysteroscopic surgery, which allowed for direct visualization of the intrauterine cavity, and endometrial biopsies were taken for further analysis. To characterize endometritis, plasma cell infiltration was assessed. Patients with ≥5 plasma cells observed in 10 high-power fields were defined as having chronic endometritis.

**Results:**

Endometritis was observed in 51.3% of the patients, totaling 172 individuals. Logistic regression analysis revealed that patients with endometrial polyps had 5.2 times higher odds of developing endometritis compared to patients without polyps (95% CI = 2.9, 9.2) (*p*-value <0.001). Similarly, patients with intrauterine adhesions had a significant increase in the odds of endometritis (OR = 4.6, 95% CI = 2.1, 10.1) (p-value <0.001).

**Conclusions:**

Treatment or removal of endometrial abnormalities through hysteroscopic procedures may help to reduce the risk of chronic endometritis and improve fertility outcomes. Further research is necessary.

## INTRODUCTION

Chronic endometritis (CE) is an inflammatory condition that is mostly characterized by presence of plasma cells in the endometrial stroma (Kaku *et al*., 2020; Xu *et al*., 2020). CE has a high prevalence in infertile women and has been associated with adverse effects on implantation rates and pregnancy outcomes in patients undergoing In-Vitro Fertilization (IVF) (Hirata *et al*., 2021). Bacterial infection is recognized as one of the leading and major risk factors for CE in infertile women (Kitaya *et al*., 2013; 2016). In these cases, the infection triggers an immune response in the endometrium, leading to the infiltration of plasma cells (D’Ippolito *et al*., 2016; Negishi *et al*., 2021). Antibiotic therapy had shown promise in improving IVF and pregnancy outcomes in patients with CE caused by bacterial infection (Cicinelli *et al*., 2015). By targeting and eliminating the bacterial infection, antibiotics can reduce the inflammatory response and improve the overall uterine environment for embryo implantation and development (Vitagliano *et al.*, 2018). However, it is important to note that bacterial infection is not the sole cause of CE. Other risk factors (infectious and non-infectious) can also contribute to the development of CE such as; sexually transmitted infections, endometrial trauma, intrauterine device use, hormonal imbalances, and immune dysregulation, among others (Kitaya *et al*., 2016).

Intrauterine abnormalities have been identified as potential risk factors that can increase the risk of CE in infertile women. Several studies have demonstrated an association between specific intrauterine abnormalities and an increased likelihood of being diagnosed with CE (Xiang *et al*., 2023; Liu *et al.*, 2019). However, the previous findings are controversial in this regard. While some studies have reported a significant association between specific intrauterine abnormalities and an increased risk of CE, others have shown conflicting results (Vitagliano *et al*., 2021). Moreover, the role of intrauterine abnormalities in etiology of CE have less been considered and there is limited evidence on this topic. Therefore, to gain a deeper understanding of the role of intrauterine abnormalities in the etiology of CE, more focused research efforts are needed. The current study aimed to investigate the association between intrauterine abnormalities, such as endometrial polyps, submucosal myomas, intrauterine adhesions, or a septate uterus and likelihood of CE in infertile women who underwent hysteroscopy at the Ayatollah Taleghani Hospital infertility center, Tehran, Iran in 2022. Expanding the evidence base in this area will help clinicians and researchers to better identify and manage the risk of CE in infertile women with intrauterine abnormalities. This would ultimately lead to improved diagnostic accuracy, personalized treatment approaches, and better reproductive outcomes for these patients.

## MATERIAL AND METHODS

### Study participants

A cross-sectional study was conducted on 335 infertile women who underwent hysteroscopic surgery at the Ayatollah Taleghani Hospital Infertility Center, affiliated with Shahid Beheshti University of Medical Sciences, Tehran, Iran in 2022. The consent form )The Royal College of Obstetricians and Gynecologists has produced guidance for gynecological services during the COVID-19 pandemic (is translated and prepared, after full explanations to the patient by Dr. Mitra Nemati, the patient will fill out the consent form in writing and will be informed about all the steps.

The current study was reviewed by board at the Shahid Beheshti University of Medical Sciences, NO.2, Arabi St., Chamran Highway, Tehran, Iran. It was corrected and revised in the Research Center of the Faculty of Medicine of Shahid Beheshti University of Medical Sciences, and finally it was registered in this center. It was able to obtain the code of ethics of the research committee. Look up details (Ethics Code; IR.SBMU.REC>1401.686).

The study included women under the age of 45 years old, who were suspected of having anatomical disorders which were diagnosed by vaginal ultrasound such as endometrial polyps, submucosal myomas, intrauterine adhesions, or a septate uterus, and who were candidates for hysteroscopy. Patients with cervical cancer, and those above the age of 45 were excluded from the study. We collected demographic and clinical data including age, infertility duration, type and cause of infertility, and signs and symptoms.

### Hysteroscopy and endometrial biopsy

All study participants underwent hysteroscopic examination using a 3-1 mm rigid hysteroscope. The uterine cavity was distended with normal saline solution at a pressure of 100 mmHg during the procedure. In all participants, endometrial biopsies with curette number 4 were performed. The retrieved biopsy samples were immediately transferred to 10% normal saline solution and then sent to the pathological laboratory within 24-48 hours for further analysis. To characterize endometritis, plasma cell infiltration was assessed. Patients with ≥5 plasma cells observed in 10 high-power fields were defined as having chronic endometritis (Puente *et al*., 2020).

### Statistical analysis

Mean and standard deviation (SD) were provided for continuous variables, while number and percent frequency were used to describe dichotomous variables. The association between each endometrial abnormality and CE was investigated using the Chi-square test. A simple logistic regression model was employed to calculate unadjusted odds ratios for each endometrial abnormality compared to patients without abnormalities. Variables with a *p*-value < 0.1 in the Wald test were included in the multiple logistic regression model to examine the adjusted odds ratios for the presence of endometrial abnormalities and chronic endometritis, while controlling for other factors. All statistical analyses were performed using Stata software, version 17.0, developed by Stata Corp in College Station, Texas, USA. A significance level of *p*-value < 0.05 was considered statistically significant.

## RESULTS

This study involved 335 patients who underwent hysteroscopy. The mean age of the participants was 37.2 years with a standard deviation (SD) of 4.6 years. It was found that 42.1% of the patients had a duration of infertility between 5 and 10 years. Among the study participants, 81.5% had primary infertility, and in 44.1% of the cases, female factors were identified as the cause of infertility. The most reported symptom was vaginal discharge, which was observed in 9.2% of the patients (Table 1).

**Table 1:** Study participants baseline characteristics.

Characteristics	N (%)
Duration of infertility (year) Less than 5 years 5-10 years Over 10 years	120 (35.8%) 141 (42.1%) 74 (22.1%)
Infertility type Primary Secondary	273 (81.5%) 62 (18.5%)
Cause of Infertility Male factor Female factor Both male and female Unknown	104 (31.0%) 148 (44.1%) 28 (8.3%) 55 (16.4%)
Symptoms and disorders AUB PID Dyspareunia Vaginal discharge	15 (4.4%) 24 (7.2%) 27 (8.0%) 31 (9.2%)
Total	335 (100%)

The overall prevalence of endometrial disorders was 79.1%, with only 70 patients (20.9%) being classified as normal. The most common abnormality observed was intrauterine disorders, followed by endometrial polyps, intrauterine adhesions, submucosal myomas, and septate uterus. Endometritis, an inflammation of the endometrium, was observed in 51.3% of the patients, totaling 172 individuals (Table 2).

**Table 2:** Prevalence of chronic endometritis in infertile women undergoing hysteroscopic surgery.

Characteristics	N (%)	N of endometritis (%)	p-value
Endometrial polyp No Yes	153 (45.7%) 182 (54.3%)	56 (36.6%) 116 (63.7%)	0.001
Submucosal myoma No Yes	309 (92.4%) 26 (7.8%)	160 (51.7%) 12 (46.1%)	0.581
Intrauterine adhesion No Yes	292 (87.2%) 43 (12.8%)	146 (50.0%) 26 (60.5%)	0.200
Septate uterus No Yes	23 (96.4%) 12 (3.6%)	168 (52.0%) 4 (33.3%)	0.204
Intrauterine disorders No Yes	70 (20.9%) 265 (79.1%)	13 (18.6%) 159 (60.0%)	0.001
Overall	335 (100%)	172 (51.3%)	---

To investigate the association between each abnormality and endometritis, a multiple logistic regression model was used. The analysis revealed that patients with endometrial polyps had 5.2 times higher odds of developing endometritis compared to patients without polyps (95% CI = 2.9, 9.2) (*p*-value < 0.001). Similarly, patients with intrauterine adhesions had a significant increase in the odds of endometritis (OR = 4.6, 95% CI = 2.1, 10.1) (*p*-value < 0.001). However, no significant association was found between endometritis and submucosal myomas or septate uterus (*p*-value > 0.05) (Table 3).

**Table 3:** Pregnancy results.

	Group A Only 2 embryos	Group B 2 selected embryos	Group C Only 3 embryos
Clinical pregnancy	94/219 (42.92%) ^*^	218/357 (61.06%) ^**^	121/208 (58.17%) ^**^
Implantation	101/479 (21.09%) ^*^	779/1901 (40.98%) ^**^	410/1109 (36.97%) ^***^
(≥2 fetuses) multiple pregnancy	11/94 (11.70%) ^*^	68/218 (31.19%) ^**^	45/121 (37.19%) ^**^
High order multiple pregnancy rate	1/94 (1.06%) ^*^	2/218 (0.92%) ^*^	17/121 (14.05%) ^**^
Miscarriage	11/94 (11.70%)	22/218 (10.09%)	15/121 (12.40%)
Ectopic pregnancy	1/94 (1.06%)	2/218 (0.92%)	1/121 (0.83%)
Delivery rate	82/219 (37.45%) ^*^	194/357 (54.34%) ^**^	105/208 (50.48%) ^**^

(^*^,^**^,^***^)Differ significantly (P < 0.05).

We also investigated diagnostic value of endometrial polyp and intrauterine adhesion. According to Table 4, sensitivity, and specificity of endometrial polyp in diagnosis of chronic endometritis was 67.4%, and 59.5%, respectively. For intrauterine adhesion the reported sensitivity and specificity was 15.1%, and 89.6%. More details are provided in Table 4.

**Table 4:** Diagnostic value of endometrial polyp and intrauterine adhesion in diagnosis of chronic endometritis in comparison to plasma cell infiltration as the gold standard.

Measure	Endometrial polyp		Intrauterine adhesion	
	Point estimate	95% confidence interval	Point estimate	95% confidence interval
Sensitivity	67.4%	59.9, 74.4	15.1%	10.1, 21.4
Specificity	59.5%	51.6, 67.1	89.6%	83.8, 93.8
ROC area	0.63	0.58, 0.69	0.52	0.49, 0.56
DOR	3.04	1.9, 4.7	1.5	0.8, 2.9
PPV	63.4%	58.6, 68.0	60.1%	46.1, 72.7
NPV	63.7%	58.0, 69.0	50.3%	48.3, 52.4

* DOR= Diagnostic Odds Ratio, PPV= Positive Predictive Value, NPV= Negative Predictive Value.

## DISCUSSION

CE is an inflammatory status associated with various reproductive challenges. It may affect the results including; infertility, pregnancy loss, recurrent implantation failures (RIF), abortions and in general in vitro fertilization (IVF) failure (Puente *et al*., 2020; Freitag *et al*., 2020). as we know, the dialogue between the embryo and the endometrium in the process of implantation is very complex and under various factors. One of these factors is chronic or/and acute endometritis. The presence of persistent inflammation in the endometrial lining of the uterus characterizes it (Smith *et al*., 2010; Kimura *et al*., 2019) .And we know that the dialogue between the embryo and the endometrium is important during implantation. This condition can result from various causes, such as bacterial or viral infections, autoimmune disorders, or previous uterine procedures (Kitaya *et al*., 2013). In the current study, we investigated the association between intrauterine abnormalities and CE in infertile women who underwent hysteroscopy. We aimed to examine whether intrauterine abnormalities, such as endometrial polyps, submucosal myomas, intrauterine adhesions, or a septate uterus, were associated with an increased likelihood of developing chronic endometritis. In the current study, the prevalence of among infertile women who underwent hysteroscopy was 51.3%. This indicates that more than half of the study participants had this inflammatory condition in their endometrial lining. Furthermore, the study revealed a significant association between CE and two specific intrauterine abnormalities, namely endometrial polyps and intrauterine adhesions. This suggests that women with endometrial polyps or intrauterine adhesions were more likely to develop CE than those without these abnormalities.

In our study, we found the prevalence of CE in infertile women undergoing hysteroscopy to be 51.3%, comparable to the prevalence reported by Kuroda *et al*. at 69.7% (Kuroda *et al*., 2022). However, it is important to acknowledge that different studies may report varying prevalence rates of CE in infertile women (Hirata *et al*., 2021; Kasius *et al*., 2011). The study conducted by Bandarian *et al*. (2023) reported a lower prevalence of CE at 21.1% in a similar population of infertile women. Several factors may contribute to the differences in prevalence rates among studies. One factor could be variations in the study populations, including differences in geographic location, ethnicity, and other demographic characteristics. Another factor is the variability in diagnostic criteria used to define chronic endometritis. The choice of diagnostic methods and thresholds for diagnosing CE can vary between studies, leading to differences in prevalence estimates. Additionally, the prevalence of CE may be influenced by the baseline characteristics of the study participants, such as the presence of intrauterine abnormalities. Studies that include a higher proportion of participants with intrauterine abnormalities may observe a higher prevalence of chronic endometritis, as these abnormalities could contribute to the development of endometrial inflammation (Kuroda *et al*., 2022). Our study found that specific intrauterine abnormalities, specifically endometrial polyps, and intrauterine adhesion, were associated with an increased risk of chronic endometritis. This finding is consistent with the results reported by Kuroda *et al*. (2022), indicating a similar association between these intrauterine abnormalities and chronic endometritis. Endometrial polyps and intrauterine adhesions can disrupt the normal architecture and function of the endometrium, potentially leading to chronic inflammation and the development of endometritis (Dreisler *et al*., 2009). These abnormalities can create a favorable environment for bacterial colonization and contribute to persistent infection and inflammation within the endometrial tissue (Dreisler *et al*., 2009). The study conducted by Bandarian *et al*. (2023), as well as our own study, demonstrated that hysteroscopic findings, including intrauterine abnormalities, can serve as suitable predictors of CE with a high diagnostic accuracy. The presence of intrauterine abnormalities detected during hysteroscopy, such as endometrial polyps, intrauterine adhesions, or other structural abnormalities, can provide important clues to the presence of chronic endometritis (Kuroda *et al*., 2022; Chen *et al*., 2016; Zolghadri *et al*., 2011). These abnormalities can serve as indicators of underlying inflammation and infection within the endometrial lining.

The current study represents one of the initial efforts to explore the association between intrauterine abnormalities and CE in infertile women. It employed the most valid criteria and analysis methods to investigate this relationship. However, it is important to interpret the findings in light of the study’s limitations. One of the main limitations of our study was its cross-sectional design, which restricts our ability to assess the impact of intrauterine abnormalities on pregnancy outcomes in infertile women. Longitudinal studies or randomized controlled trials would provide more definitive evidence regarding the relationship between intrauterine abnormalities, chronic endometritis, and fertility outcomes. Another limitation to consider was the diagnostic criteria used for defining chronic endometritis. In our study, we utilized the presence of plasma cell-positive cells per 10 nonoverlapping random stromal areas as the criterion for diagnosing chronic endometritis. However, it is important to note that different studies may employ varying diagnostic criteria, which could influence the reported prevalence and associations.

In conclusion, The association between intrauterine abnormalities and CE highlights the importance of identifying and addressing these abnormalities in infertile women. Treating or removing these intrauterine abnormalities through hysteroscopic procedures may help reduce the risk of CE and improve fertility outcomes. Further research is warranted to investigate the underlying mechanisms linking intrauterine abnormalities, chronic endometritis, and infertility. Additionally, prospective studies evaluating the effectiveness of interventions targeting these intrauterine abnormalities in reducing the risk of CE and improving reproductive outcomes would be valuable in guiding clinical practice.
